# *N*-(2-mercaptopropionyl)-glycine enhances in vitro pig embryo production and reduces oxidative stress

**DOI:** 10.1038/s41598-020-75442-6

**Published:** 2020-10-29

**Authors:** J. M. Cambra, C. A. Martinez, H. Rodriguez-Martinez, E. A. Martinez, C. Cuello, M. A. Gil

**Affiliations:** 1 Department of Medicine and Animal Surgery, Faculty of Veterinary Medicine, International Excellence Campus for Higher Education and Research “Campus Mare Nostrum”, University of Murcia, 30100 Murcia, Spain; 2 Institute for Biomedical Research of Murcia (IMIB-Arrixaca), Campus de Ciencias de la Salud, Carretera Buenavista s/n, 30120 El Palmar, Murcia, Spain; 3 Department of Biomedical and Clinical Sciences (BKV), BKH/Obstetrics and Gynaecology, Faculty of Medicine and Health Sciences, Linköping University, 58185 Linköping, Sweden

**Keywords:** Animal biotechnology, Embryology, Animal breeding

## Abstract

This study evaluated the effects of different concentrations (1, 10, 25, 50, and 100 µM) of the antioxidant *N*-(2-mercaptopropionyl)-glycine (NMPG), during the culture of in vitro-fertilized porcine oocytes. While the highest concentrations of NMPG (50 and 100 µM) were toxic to the developing embryos during the first two days of culture, 25 µM NMPG achieved cleavage rates that were similar to those achieved by the control but did not sustain blastocyst production by Day 7 of culture. Compared to the control culture medium, the culture medium supplemented with 10 µM NMPG increased (*P* < 0.05) the rates of blastocyst formation, decreased (*P* < 0.05) the intracellular levels of reactive oxygen substances, and downregulated (*P* < 0.05) the expression of the oxidative stress related gene GPX1. In conclusion, these results demonstrated that supplementation of porcine embryo culture medium with 10 µM NMPG can attenuate oxidative stress and increase the yield of in vitro production of blastocysts.

## Introduction

In vitro production of porcine embryos has numerous applications in both livestock production and biomedical research fields. Over the past 20 years, many advances have been made toward improving the culture of in vitro-produced porcine embryos^1^. Yet, the quality of the embryos produced with this technique still differs greatly from the quality of the embryos obtained in vivo^2^. A key method to overcome this hurdle has been to identify an optimal culture milieu that simulates, to the extent possible, the in vivo embryonic environment. One of the main differences between the in vitro and in vivo embryonic developmental conditions is related to the prevalence of oxidative stress. Under in vitro culture conditions, several exogenous factors increase the production of reactive oxygen species (ROS) over physiological limits, such as exposure to light^3,4^, an oxygen-rich atmosphere^5^, or the burden of different compounds that are present in the embryo culture media, such as metallic cations, amine oxidase from the serum or high concentrations of glucose^6^. Physiologically, ROS are neutralized by antioxidants, and glutathione (GSH) is one of the main nonenzymatic antioxidants in animal cells^7^. However, an excessive increase in ROS during in vitro culture disrupts the redox balance; altering different cell molecules, such as lipids, proteins or nucleic acids, and leading to mitochondrial dysfunction, ATP depletion, apoptosis and finally, the fragmentation of the embryo reviewed by Takahashi^8^. One of the most widely used methods to maintain the intra- and extracellular redox balance of in vitro-produced porcine embryos has been the supplementation of the media with different types of antioxidants, such as Trolox^9^, γ-tocotrienol^10^, C-phycocyanin^11^, carboxyethylgermanium sesquioxide^12^ or ascorbic acid^13^. *N*-(2-mercaptopropionyl)-glycine (NMPG), also known as tiopronin, is a glycine-derived diffusible antioxidant that acts as a scavenger of free radicals. In its chemical structure, NMPG includes a thiol or a sulfhydryl group, with the ability to donate protons or electrons; making NMPG a relatively specific compound for the reduction of hydroxyl and peroxide type radicals^14^. Different studies^15–17^ have demonstrated the capacity of NMPG to reduce the deleterious effects of ROS in injuries caused by cardiac ischemia. This product has also been used in vitro to counteract high oxidative stress conditions in rat pheochromocytoma (PC12) cells^18^, skeletal muscle fibers^19^ and human prostate cancer cells^20^.

Despite these properties, the effects of NMPG during in vitro embryo production have not yet been described in any species. Therefore, the aim of our study was to evaluate the effects of the addition of NMPG to the embryo culture medium on the development, quality and oxidative balance of porcine embryos.

## Results

A representative subset of presumptive zygotes (N = 403) from each replicate in both experiments was fixed and stained at 18 h post-insemination to assess in vitro fertilization parameters. In both experiments, the results were similar among the replicates and to those previously reported by our laboratory. The overall rates of maturation, penetration, monospermy and fertilization efficiency were 93.8 ± 3.7%, 72.3 ± 9.4%, 60.0 ± 8.4% and 40.1 ± 3.3%, respectively.

### Experiment 1: Effects of different concentrations of NMPG during culture on embryonic development

Since there are no previous reports about the effects of NMPG on embryos, an initial dose-related-effect experiment was performed to establish the best concentration for use during in vitro culture. After in vitro fertilization, presumptive zygotes (N = 1998; 3 replicates) were cultured with different concentrations of NMPG: 0 (Control), 1, 10, 25, 50, and 100 µM. The embryonic developmental stages were evaluated at Days 2, 5, 6 and 7 of culture.

**Figure 1:**
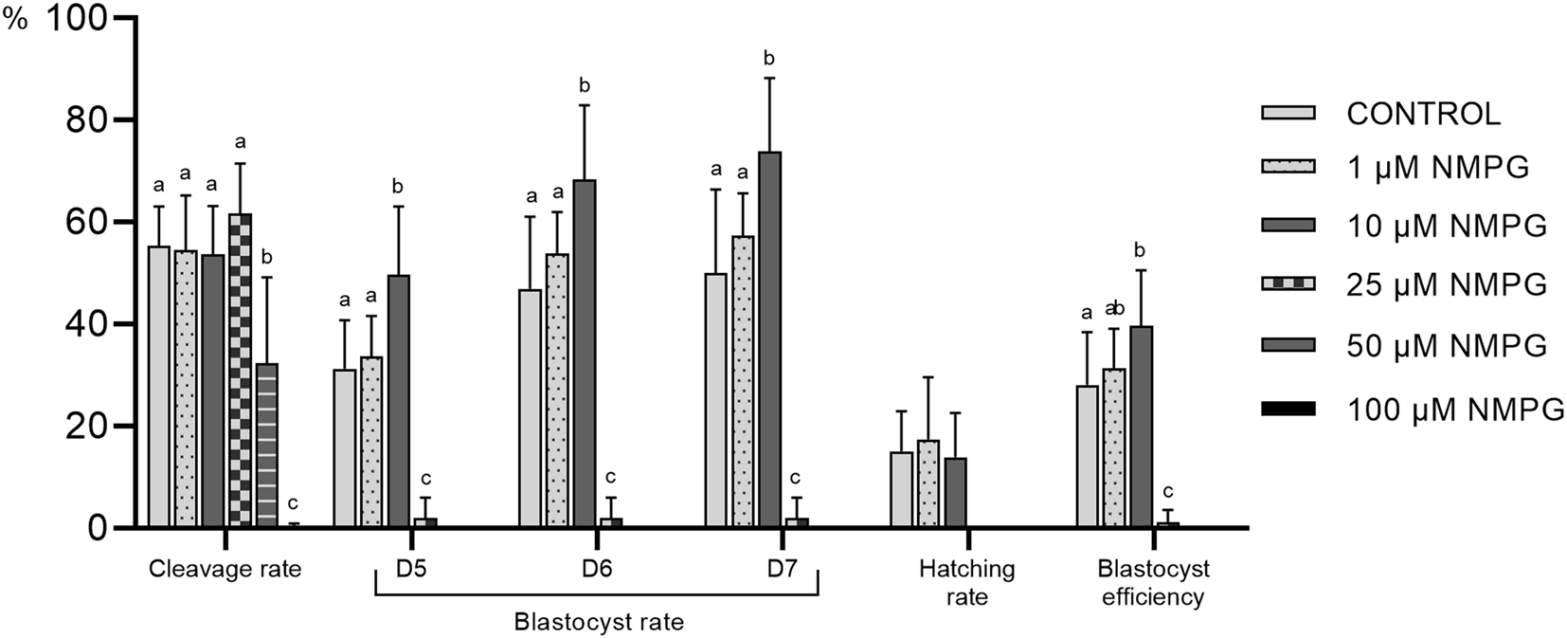
Developmental competence of in vitro-fertilized oocytes cultured in embryo culture medium supplemented with 0, (Control; N = 399), 1 µM (N = 359), 10 µM (N = 403), 25 µM (N = 161), 50 µM (N = 236) and 100 µM (N = 440) NMPG. The presumed zygotes were cultured in NCSU-23 with 0.3% polyvinyl alcohol and 100 ng/mL platelet factor 4 for seven days. The data are expressed as the mean ± SD (three replicates). Within the same variable, values with different superscript letters (a**–**c) indicate significant differences (*P* < 0.05).

The embryonic development data are listed in Fig. 1. The concentrations of 50 and 100 µM NMPG showed different degrees of toxicity during the first two days of culture, which was reflected in a decreased (*P* < 0.05) cleavage rate, compared to the other concentrations. None of the cleaved embryos from these two groups reached the blastocyst stage. Although the 25 µM NMPG group had a similar cleavage rate compared to the control group and the 1 and 10 µM NMPG groups, only 2% of the cleaved embryos were able to develop to the blastocyst stage. The embryos cultured with 1 µM NMPG reached the blastocyst stage at rates similar to the control embryos. Compared with the control, the supplementation of the embryo culture with 10 µM NMPG enhanced (*P* < 0.05) the blastocyst formation rates on Days 5, 6, and 7 and the blastocyst efficiency. There were no differences in the hatching rates among the 1 µM NMPG, 10 µM NMPG and control groups (range 14.0 ± 8.6% to 17.4 ± 12.2%).

### Experiment 2: Effects of 10 µM NMPG during culture on the quality and oxidative balance of embryos

Based on the results obtained in experiment 1, after in vitro fertilization, a total of 4310 presumptive zygotes were randomly divided into two groups: the control group (zygotes cultured in the absence of NMPG) and the 10 µM NMPG group (zygotes cultured with 10 µM NMPG), in a total of 9 replicates. Embryonic development variables were assessed at days 2, 5, 6 and 7 of culture. The Day 7 blastocysts were used for TCN count (N = 356), differential staining (N = 29), ROS and GSH measurement (N = 98) and gene expression analyses (N = 160).

#### In vitro* embryo culture variables*

**Figure 2:**
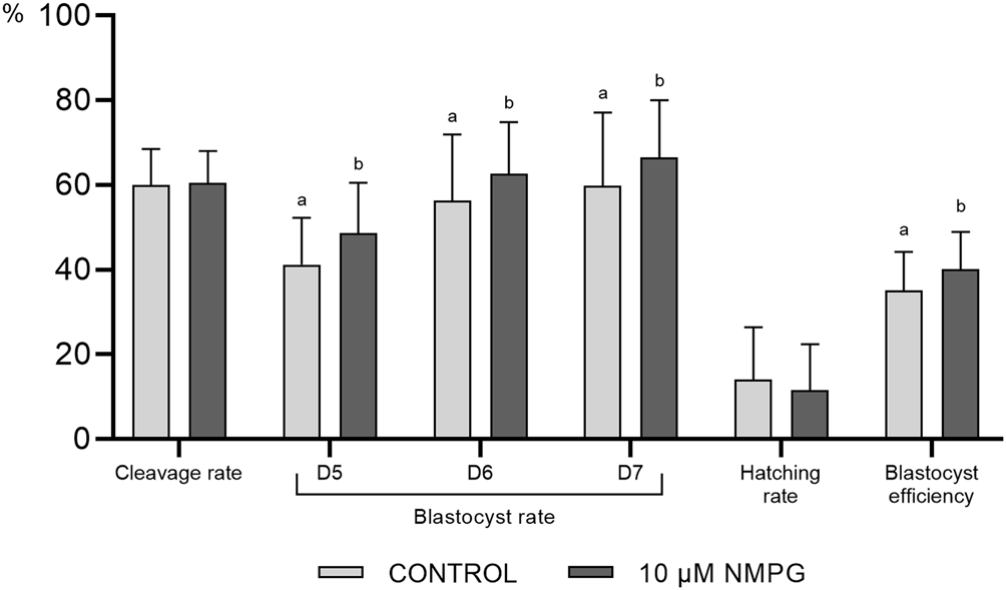
Developmental competence of in vitro-fertilized oocytes cultured in embryo culture medium supplemented with 0 (Control; N = 1874) or 10 µM (N = 2023) NMPG. The presumed zygotes were cultured in NCSU-23 with 0.3% polyvinyl alcohol and 100 ng/mL platelet factor 4 for seven days. The data are expressed as the mean ± SD (nine replicates). Within the same variable, values with different superscript letters (a, b) indicate significant differences (*P* < 0.05).

The variables accounted for embryonic development were similar to those observed in Experiment 1 (Fig. 2). Compared with the control, the addition of 10 µM NMPG to the culture medium did not affect the cleavage and hatching rates and increased (*P* < 0.05) the blastocyst formation rates at days 5, 6 and 7 and the blastocyst efficiency rate.

#### Blastocysts TCN and differential staining

**Figure 3:**
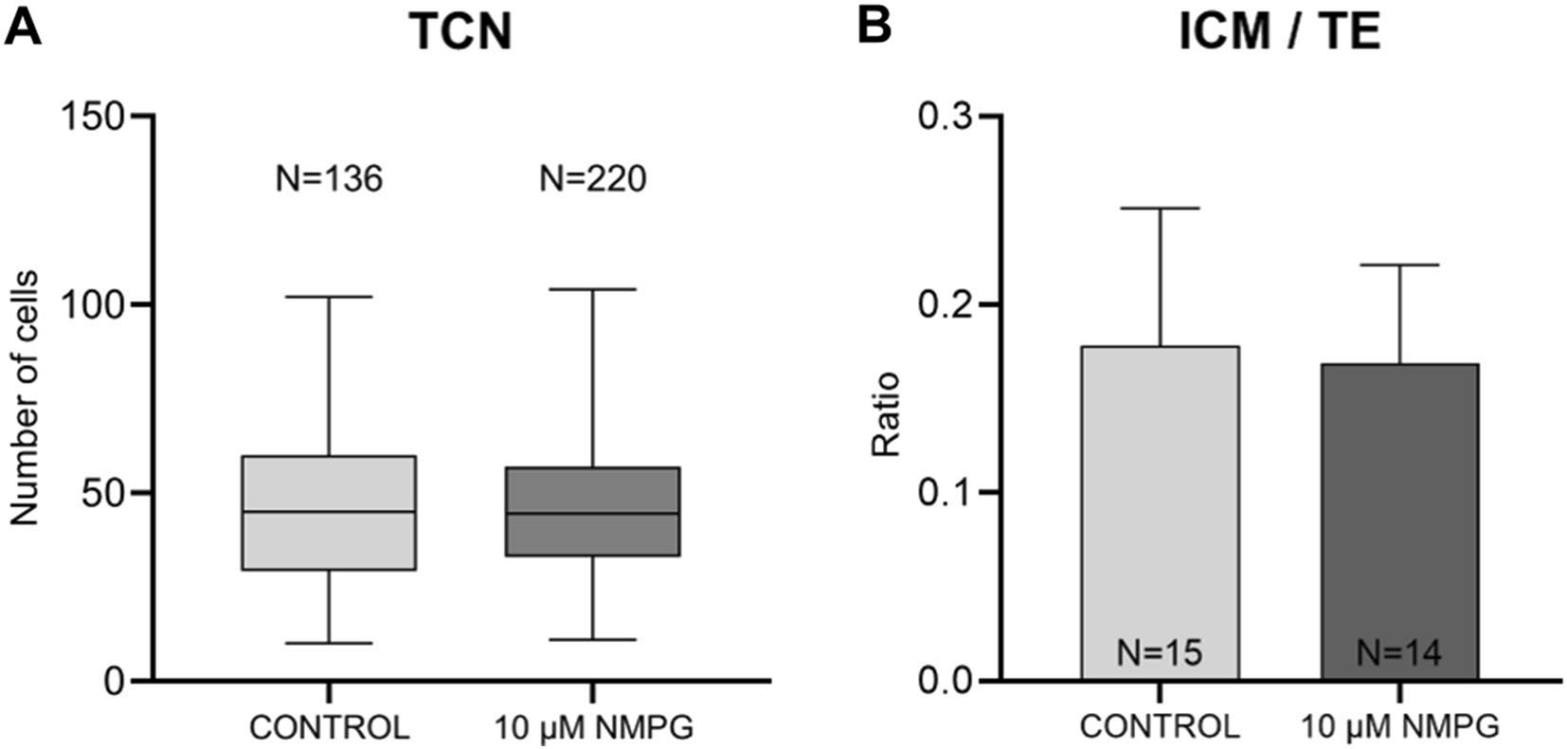
Total cell number (TCN) of blastocysts obtained on Day 7 that were produced in embryo culture medium supplemented with 0 (Control; N = 136) or 10 µM NMPG (N = 220) (**A**). Ratio of inner cell mass (ICM) cells to trophoectoderm (TE) cells of the Day 7 blastocysts that were produced in embryo culture medium supplemented with 0 (Control; N = 15) or 10 µM NMPG (N = 14). The data are expressed as the mean ± SD (**B**).

There were no significant differences in the TCN of blastocysts between the 10 µM NMPG and control groups (46.6 ± 19.2 and 46.6 ± 20.6, respectively) (Fig. 3A). Similarly, supplementation with 10 µM NMPG did not affect the ICM/TE ratio (0.17 ± 0.05) compared to the controls (0.18 ± 0.07) (Fig. 3B).

#### Intracellular levels of ROS and GSH

**Figure 4:**
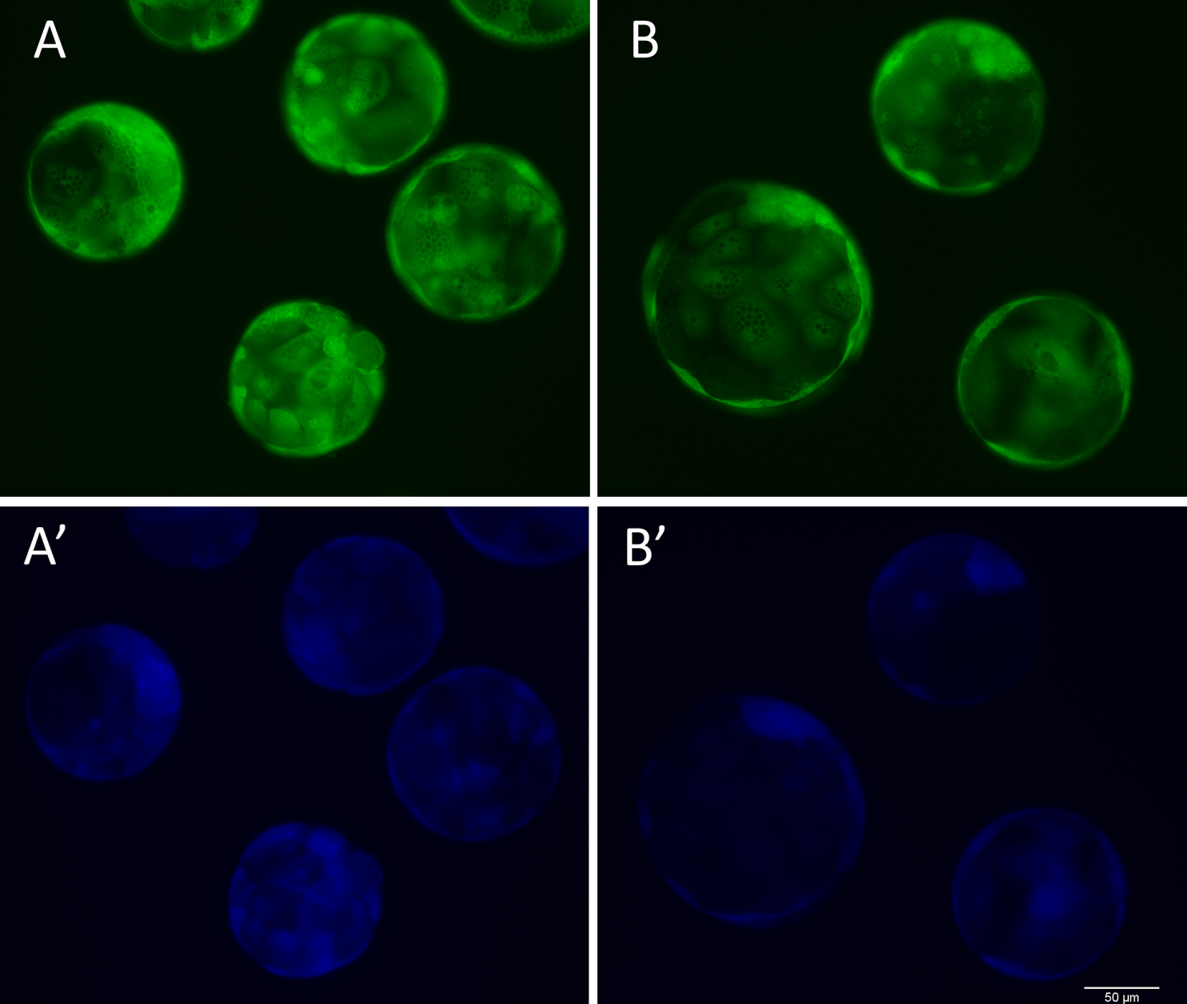
Representative images of Day 7 blastocysts stained with H2DCFDA or CellTracker Blue. The green and blue fluorescence indicates the relative ROS and GSH contents, respectively, in the Control (**A** and **A**′) and 10 µM NMPG (**B** and **B**′) groups.

The blastocysts produced in medium supplemented with 10 µM NMPG showed lower (*P* < 0.05) ROS contents (0.89 ± 0.19) and tended (*P* = 0.07) to display lower GSH contents (0.86 ± 0.26) compared to the controls blastocysts (0.98 ± 0.15 and 0.96 ± 0.23, respectively). Representative fluorescence microscopy images are depicted in Fig. 4.

#### Gene expression results

**Figure 5:**
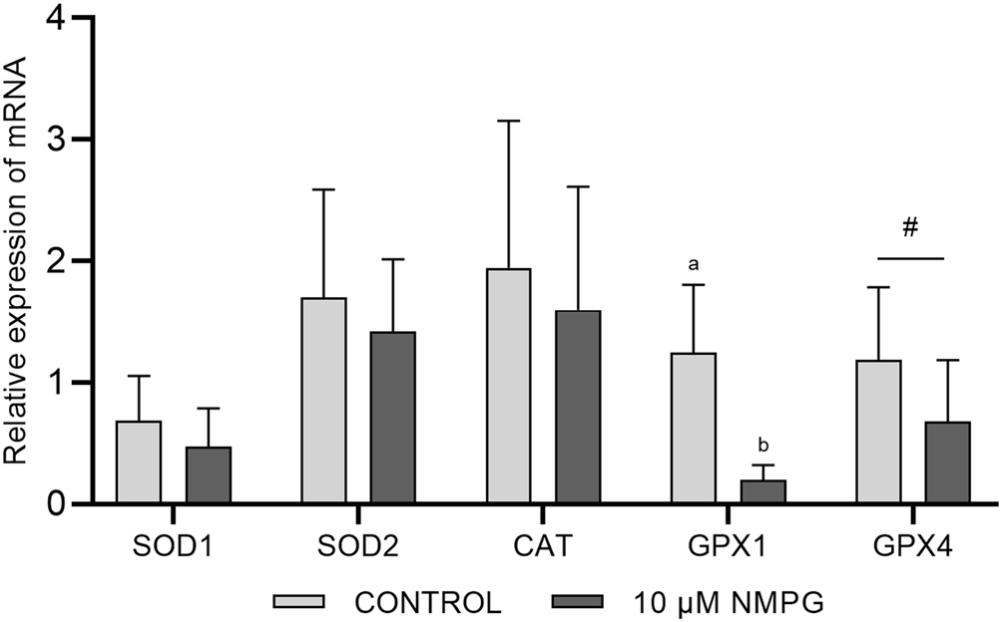
Relative transcript abundance of oxidative stress-related genes in Day 7 blastocysts that were produced in embryo culture medium supplemented with 0 (Control) or 10 µM NMPG. The data are expressed as the mean ± SD. Within the same gene, values with different superscript letters (a, b) indicate significant differences (*P* < 0.001). # *P* = 0.09 indicates a nonsignificant trend between the Control and 10 µM NMPG groups.

**Figure 6:**
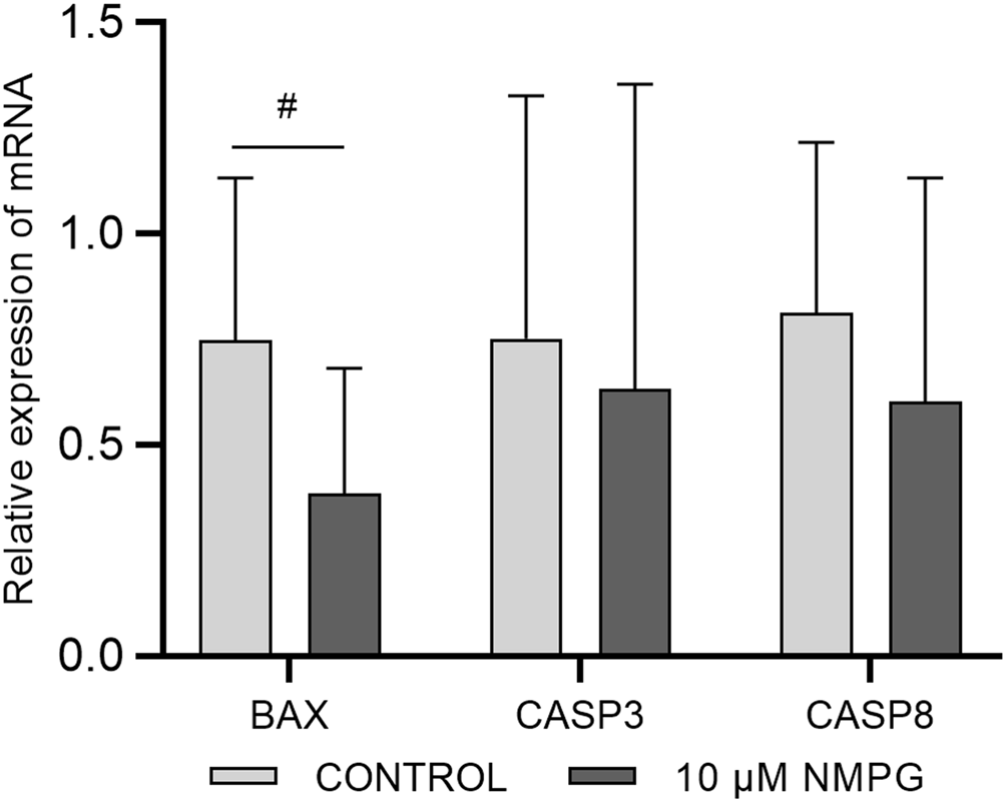
Relative transcript abundance of apoptosis-related genes in Day 7 blastocysts that were produced in embryo culture medium supplemented with 0 (Control) or 10 µM NMPG. The data are expressed as the mean ± SD. #*P* = 0.06 indicates a different trend between the Control and 10 µM NMPG groups.

From the total number of oxidative stress-related genes that were analyzed (Fig. 5), only GPX1 was significantly (*P* < 0.01) downregulated in the 10 µM NMPG group. The expression of GPX4 showed a decreasing trend (*P* = 0.09) in the experimental group compared to the control group. Other apoptosis-related genes that were analyzed (Fig. 6) did not show significant differences between the groups; only BAX gene expression showed a decreasing trend (*P* = 0.06) in the 10 µM NMPG group compared with the control group. The expression of the antiapoptotic gene BCL2 was also analyzed, however due to the low expression observed in different samples, these results were finally discarded.

## Discussion

The results of the present study are novel, showing that the supplementation of embryo culture medium with the antioxidant NMPG at a concentration of 10 µM enhances the blastocyst formation rates, reduces the intracellular ROS content and modifies the gene expression of some oxidative stress-related genes.

The experiments included in this study were performed in a defined embryo culture medium. This medium supports the development of pig blastocysts in the absence of BSA, as previously demonstrated in our laboratory^21^. The use of this defined media allowed us to more accurately evaluate the effects of NMPG supplementation and to avoid any possible interaction with undefined compounds, as has been previously reported using other additives in culture medium^22,23^.

Since no previous reports have been published that examine the effects of NMPG on embryos, an initial experiment was designed to evaluate the optimal concentration of this antioxidant in porcine embryo culture. In that experiment, a range of concentrations was tested.

Supplementation with 50 and 100 µM NMPG was shown to be toxic, significantly reducing the rate of cleaved embryos in a dose-dependent manner, and jeopardizing blastocyst production. Furthermore, although the embryos cultured with 25 µM NMPG were able to cleave at similar as controls, blastocyst formation was drastically reduced. This fact further points to this concentration of NMPG as embryo toxic. ROS regulate different cellular pathways related to proliferation^24^ and differentiation^25^, among others, and act as intracellular messengers. A substantial decrease in ROS levels may alter normal cellular function, which could explain why the high concentrations of the antioxidant used in our experiments had deleterious effects on our embryos.

On the other hand, 1 µM-NMPG did not influence embryonic development, but 10 µM NMPG significantly increased the number of blastocysts formed, although neither cleavage nor hatching rates did not improve compared to controls.

In pigs, it has been reported that supplementation with different antioxidants, such as crocin^26^, sphingosine-1-phosphate^27^ or melatonin^28^, during in vitro maturation culture improved the cleavage rates. Nevertheless, when supplementation was performed exclusively during embryo culture, the cleavage rate was not affected^9,11^. These findings are consistent with our results, since we did not observe improvements in the cleavage rate at Day 2 after any of the NMPG concentrations used.

Surprisingly, the concentration that showed significant improvements in blastocyst formation (10 µM NMPG) laid between 10- and 1000-fold lower than those levels used when culturing different cell lines^18–20,29,30^. A possible explanation for the greater sensitivity of embryos to NMPG, compared to homogeneous single cell lines, could be that the requirement for ROS may be greater in poorly differentiated cells, such as preimplantation embryos, since they need these substances as molecular signals in the pathways related to cell differentiation and proliferation described above. Alternatively, the fact that embryos differentiate early in at least two very different cell types (trophoblast vs. inner cell mass) might be relevant compared to single-cell type cultures. Therefore, a large reduction in ROS caused by NMPG limits embryonic development; however, in cells with a higher degree of differentiation, where the activity of these pathways is lower, higher concentrations of NMPG, and consequently lower concentrations of ROS, can be tolerated without major detrimental effects.

The capability of NMPG as an ROS scavenger was studied by Valachova^14^. These authors described that this compound is an effective scavenger of hydroxyl and particularly peroxid-type radicals. This type of free radical mainly results from the peroxidation of lipids^31^, and the pig being a particularly sensitive species due to the high lipid contents in embryos^32^. The activity of NMPG in scavenging this type of radical may explain the improvement in the in vitro blastocyst production observed in the 10 µM NMPG supplementation group. In Experiment 2, the addition of 10 µM NMPG decreased the ROS content of the Day 7 blastocysts compared to that of the control blastocysts. This observation is consistent with the NMPG scavenger properties described for NMPG, which decrease the oxidative stress of the embryos and, consequently, improve their development capacity.

Usually, supplementation of embryo culture media with antioxidants is associated not only with improvements in embryo production rates, but also with enhancing embryo quality^12,33^. In our study, the increased blastocyst rates observed after supplementation with 10 µM NMPG were not associated with an improvement in embryonic quality in terms of TCN or the ICM/TE ratio. A reduced expression of the apoptosis-related genes can be correlated with improvements in embryonic quality, however, among the genes analyzed, only a downward trend in the BAX gene was observed in the group supplemented with 10 µM of NMPG, remaining the expression of CASP3 and CASP8 genes unaltered. Unfortunately, the expression of the antiapoptotic gene BCL2 could not be analyzed due to its low expression in the blastocysts produced in vitro.

To examine if the beneficial effect of NMPG on in vitro embryo culture was related to changes in oxidative stress management, we analyzed the transcript levels of target genes encoding the most common enzymatic antioxidants. We included both superoxide dismutases, SOD1 (also known as Cu/Zn-SOD and located in the cytoplasm) and SOD2 (also known as Mn-SOD and located in the mitochondria), catalase (CAT) and two glutathione peroxidases (GPX1 and GPX4). These enzymes act in an orchestrated way and maintain a low concentration of radicals through different mechanisms of action^34^. It has been reported that cells are able to increase the expression of these enzymes when the concentration of oxygen radicals increases as a mechanism to counteract oxidative stress^35,36^. Similarly, several studies reported that the expression of these genes was downregulated when the oxygen concentrations during embryo culture were reduced and, thus, the ROS concentrations were low^37–39^. On the other hand, when embryos were exposed to increased ROS levels, these genes were upregulated^40,41^. In our experiment, only the expression of the glutathione peroxidase genes was modified, and compared to the control blastocysts, GPX1 expression was significantly downregulated in the 10 µM NMPG blastocysts, and GPX4 showed a similar trend. These results suggest an improvement in the oxidative balance during embryo culture when NMPG was present in the medium. Conversely, SOD1, SOD2 and CAT were not affected. This result could occur due to the different types of free radicals on which each enzyme acts. As mentioned above, the free radicals targeted by NMPG are specifically peroxide-type radicals, which explains why the main gene affected by NMPG belongs to the GPX family. The GPX enzymes act by detoxifying these free radicals, oxidizing two molecules of GSH, whereas SOD enzymes target the superoxide anion radical (O_2_^−^) and catalase targets hydrogen peroxide (H_2_O_2_). In addition, this fact could also explain the observed tendency of lower GSH contents in the NMPG-treated blastocysts, which could suggest that NMPG performs functions similar to GSH and therefore the requirement of these blastocysts for GSH is lower.

Additionally, NMPG reduces hypoxia-inducible factor-1α (HIF-1α) degradation^29,30^. The HIF complex regulates the pluripotency status and proliferation of embryonic stem cells^42–46^. Therefore, NMPG might also improve embryo development in vitro by increasing the levels of HIF-1α.

Moreover, the genes affecting glucose metabolism that are controlled by HIF lead to a switch in the type of glucose metabolism, promoting glycolysis and lactate production instead of oxidative phosphorylation and oxygen consumption^47^. Glycolytic metabolism does not consume oxygen; therefore, ROS production is reduced, which has a positive effect on bovine embryo culture^48^. In addition, this glycolytic metabolism generates an accumulation of glycolytic intermediates in the embryos that could provide advantages for their further development^49^. This HIF-related mechanism, together with the mechanisms reported in our study, could explain the increased rates of embryo production and the lower ROS levels observed in the blastocysts in our study. More research is needed to elucidate the mechanisms of action for NMPG on embryos.

In conclusion, the addition of 10 µM NMPG to porcine embryo culture medium improves the production of blastocysts, reduces the blastocyst levels of ROS, and modifies the expression of the GPX1 gene that regulate the oxidative balance.

## Methods

### Reagents

Unless stated otherwise, all the chemicals used in this study were obtained from Sigma-Aldrich Co. (Alcobendas, Madrid, Spain).

### Cumulus-oocyte complex (COC) collection and in vitro maturation

Ovaries from prepubertal gilts were collected in a local abattoir (El Pozo S.A., Murcia, Spain) and transported in 0.9 mg/mL NaCl with 70 µg/mL kanamycin at 35 °C. The COCs were obtained from medium-sized follicles, 3 to 6 mm in diameter, and sectioned with a surgical blade in Tyrode's lactate medium supplemented with 10 mM HEPES and 0.1 mg/mL polyvinyl alcohol (TL-HEPES-PVA)^50^. The COCs with dark and granulated cytoplasms and several compact cumulus cell layers were washed three times in IVM medium consisting of tissue culture medium (TCM) 199 (Gibco Life Technologies S.A., Barcelona, Spain) supplemented with 0.55 mM glucose, 0.9 mM sodium pyruvate, 75 µg/mL penicillin, 50 µg/mL streptomycin, 1 mg/mL PVA, 0.57 mM cysteine and 10 ng/mL epidermal growth factor. Groups of 70 to 80 COCs were matured in 500 µL of pre-equilibrated IVM medium supplemented with 10 IU eCG (Folligon, Intervet International B.V., Boxxmeer, the Netherlands) and 10 IU hCG (VeterinCorion, Divasa Farmavic, S.A., Barcelona, Spain) for 22 h. Then, the groups of COCs were incubated for an additional 22-h period in the same medium without hormones. All the incubations were performed in humidified atmosphere air with 5% CO_2_ at 38.5 °C under an oil overlay.

### In vitro fertilization

After the maturation period, the cumulus cells were removed from the oocytes by vortexing at 1660 rounds/min for 2 min in 300 µL of TL-HEPES-PVA supplemented with 0.1 mg/mL hyaluronidase. The denuded oocytes were then washed three times in IVM medium and three times in IVF medium, which consisted of Tris-buffered medium^51^ supplemented with 2 mM caffeine and 0.2 mg/mL BSA. Groups of 40 oocytes that have been previously subjected to in vitro maturation were placed in 50-µL drops of IVF medium and coincubated with frozen-thawed spermatozoa prepared as described by Gil et al.^52^. For each replicate, two semen straws were thawed at 37 °C for 20 s, and 200 µL of spermatozoa from both straws was pooled, washed three times in Dulbecco’s phosphate-buffered solution (PBS, Gibco, Grand Island, NY) supplemented with 4 mg/mL BSA, and pelleted at 1900×*g* for 3 min. The resulting pellet was resuspended in 1 mL of IVF medium, quantified with a Sperm Cell Counter NucleoCounter SP-100 (ChemoMetec, Allerød, Frederiksborg, Denmark), adequately re-suspended to 2.4 × 10^6^ spermatozoa/mL, and finally added to the IVF drop containing the oocytes. Thus, each oocyte was exposed to a total of 3000 spermatozoa in a humidified atmosphere air with 5% CO_2_ at 38.5 °C under an overlay of paraffin oil, for 5 h^53^.

### Assessment of in vitro fertilization parameters

Presumptive zygotes were fixed 18 h after insemination in a PBS solution with 0.5% (v/v) glutaraldehyde for 30 min. The fixed presumptive zygotes were placed in 2-µL drops of Vectashield (Vector, Burlingame, CA, USA) containing 10 mg/mL Hoechst 33342 on a slide and covered with a coverslip. The fixed presumptive zygotes were observed under a fluorescence microscope (excitation filter 330 to 380 nm) at 400× magnification.

The oocytes were considered immature when the chromatin was enclosed in a nuclear membrane or condensed in metaphase I, whereas they were classified as mature but not penetrated when the chromatin was organized in metaphase and the first polar body was visible (metaphase II). The oocytes were considered penetrated when at least two pronuclei were visible in the cytoplasm. In addition, when there were only two pronuclei, the monospermic status was confirmed. The maturation rate was defined as the number of oocytes in metaphase II and the number of oocytes with at least two pronuclei relative to the total number of oocytes stained. The subset of penetrated oocytes relative to the mature oocytes was defined as the penetration rate. The monospermic rate was calculated as the number of monospermic oocytes relative to the number of penetrated oocytes. The efficiency of fertilization was calculated as the percentage of monospermic oocytes over the total number of presumptive zygotes.

### Embryo culture

The medium used for embryo culture was North Carolina State University (NCSU) 23^54^ supplemented with 0.3 mg/mL PVA and 100 ng/mL platelet factor 4^21^. After gamete coculture, the presumptive zygotes were transferred to pre-equilibrated culture medium, and the spermatozoa attached to the zona pellucida were removed by mechanical pipetting. After washing, the zygotes were placed in four-well dishes with 500 µL of glucose-free culture medium supplemented with 0.3 mM sodium pyruvate and 4.5 mM lactate during the first two days of the culture period. For the next 5 days before the completion of the culture period, the embryos were placed in fresh culture medium with 5.5 mM glucose instead of sodium pyruvate and lactate.

### Evaluation of embryonic development

Embryo development was assessed considering rates of embryo cleavage, blastocysts at Days 5, 6 and 7, hatching rate and blastocysts efficiency. Embryo cleavage rate was defined as the number of embryos that reached the 2- to 4-cell stage at Day 2 of culture relative to the total number of oocytes inseminated. The blastocyst rates at Days 5, 6 and 7 of culture were defined as the number of embryos that developed to the blastocyst stage at these days relative to the number of cleaved embryos at day 2. The hatching rate was calculated as the number of hatching or hatched blastocysts at Day 7 relative to the total number of blastocysts on the same day. Finally, the blastocyst efficiency was defined as the percentage of presumptive zygotes that reached the blastocyst stage by Day 7.

### Embryo total cell number (TCN)

To count the total cell number (TCN), day 7 blastocysts were fixed for 30 min in PBS solution with 4% (v/v) paraformaldehyde. Then, the blastocysts were washed twice in PBS with 0.3 mg/mL BSA and stored at 4 °C until staining. For nuclear staining, the fixed blastocysts were placed in 2-µL droplets of Vectashield (Vector, Burlingame, CA, USA) containing 10 mg/mL Hoechst 33342 on a slide and covered with a coverslip. The stained blastocysts were observed with the excitation filter (330 to 380 nm) of a fluorescence microscope, and the number of nuclei per blastocyst that displayed blue fluorescence was counted and expressed as the TCN.

### Differential embryo staining

Inner cell mass (ICM) and trophectoderm (TE) cell staining were performed as previously described^55^ with minor modifications. First, day 7 blastocysts were fixed as described above. The fixed blastocysts were permeabilized in PBS with 1.5% (v/v) Triton X-100 and 0.15% (v/v) Tween 20 for 15 h at 4 °C and then washed three times for two minutes with washing solution (PBS with 0.3 mg/mL BSA). The blastocysts were denatured in a two-step sequential incubation consisting of an initial incubation in a 2 N HCl solution for 20 min followed by a second incubation in a 100 mM Tris solution for 10 min, both at room temperature. Then, the blastocysts were washed again three times for 2 min and blocked in a solution of PBS with 1 mg/mL BSA, 10% (v/v) normal donkey serum and 0.05% (v/v) Tween 20 for 5 h at 4 °C. After that, the blastocysts were washed and incubated for 1.5 days at 4 °C in a 1:200 solution of the ready-to-use primary CDX2 antibody (Biogenex, San Ramon, USA) in a commercial antibody diluent (Biogenex, San Ramon, USA). The expression of this protein is restricted to the TE cells in the preimplantation stages of porcine embryos^56^. A total of 8 blastocysts were incubated in the commercial diluent without anti-CDX2, serving as negative controls. After washing, the blastocysts were finally incubated with the secondary antibody (donkey anti-mouse IgG-Alexa Fluor 568, Invitrogen, Rockford, USA) diluted 1:1000 in blocking solution for 30 min at room temperature. To evaluate the cell number, the stained blastocysts were placed in 2 µL of 10 mg/mL Hoechst 33342 Vectashield solution and observed by fluorescence microscopy with 536 nm and 330 to 380 nm excitation filters. After merging both images, the purple and blue nuclei were used to identify TE and ICM cells, respectively. The ICM/TE ratio was calculated for each blastocyst as the number of ICM cells relative to the number of TE cells.

### Measurement of intracellular reactive oxygen species (ROS) and glutathione (GSH) levels

Intracellular levels of ROS and GSH were measured in Day 7 blastocysts as previously described^13^. H2DCFDA (2′,7′-dichlorodihydrofluorescein diacetate; Invitrogen, Thermo Fisher Scientific, Massachusetts, USA) was used to detect the ROS levels, while CellTracker Blue (4-chloromethyl-6.8-difluoro-7-hydroxycoumarin; CMF2HC; Invitrogen) was used to detect the GSH levels. Briefly, the blastocysts were washed in TL-HEPES-PVA and incubated in this medium supplemented with 10 µM H2DCFDA and 10 µM CellTracker Blue in the dark for 30 min at 38.5 °C. After incubation, groups of 5 blastocysts were washed three times with TL-HEPES-PVA and placed in 5-µL droplets of the same medium on glass slides. The fluorescence emission was immediately observed using a fluorescence microscope (excitation filters of 460 nm for ROS and 370 nm for GSH), and images of the fluorescent blastocysts were recorded with a digital camera. The fluorescence values of each embryo image were analyzed using ImageJ software (Version 1.51 h; National Institutes of Health, Bethesda, MD, USA) and calculated using the corrected total cell fluorescence (CTCF) according to the following equation: Integrated Density—(Area of selected embryo x Mean background fluorescence)^57^. The CTCF of the control group blastocysts was set to 1, and the ROS and GSH contents of the experimental group were calculated with respect to this value.

### RNA isolation, cDNA synthesis and quantitative PCR

After culture, the Day 7 blastocysts were washed three times with PBS and placed in 1.5-mL RNase-free Eppendorf tubes in pools of 10. A total of 8 biological replicates were collected from each experimental group. The RNA was extracted from each pool using the RNeasy Plus Micro Kit (Qiagen, Hilden, Germany) according to the manufacturer’s instructions. The RNA obtained was used for the synthesis of complementary DNA with the Maxima H Minus First Strand cDNA Synthesis Kit (Thermo Fisher Scientific Waltham, Massachusetts, USA) at 25 °C for 10 min and 50 °C for 15 min followed by 85 °C for 5 min in an Eppendorf Mastercycler.

The primers (Table 1) for 5 oxidative stress-related genes (SOD1, SOD2, CAT, GPX1 and GPX4) and 3 apoptosis-related genes (BAX, CASP3 and CASP8) were designed with Primer3Plus software^58^. The primer efficiencies ranged from 88.5 to 105.7, and these values were obtained according to the equation E = 10^[−1/slope]^, calculating the slope of the standard curve generated from 5 serial dilutions of extra cDNA.

**Table 1:** Primer sequences used for qPCR and amplicon sizes.

Gene symbol	GENE name	Forward (5′ → 3′)	Reverse (5′ → 3′)	Size	Accession number
SOD1	Superoxide dismutase 1	GGATCAAGAGAGGCACGTTG	CTGCCCAAGTCATCTGGTTT	159	NM_001190422.1
SOD2	Superoxide dismutase 2	GATTGCCGCTTGTTCTAACC	CGCTCAGTTACATTCTCCCAGT	168	NM_214127.2
CAT	Catalase	CACAGCGAATACCCTCTTATCC	ACGGAAGGGACAGTTCACAG	228	NM_214301.2
GPX1	Glutathione peroxidase 1	CGGGACTACACCCAGATGA	GCCATTCACCTCACACTTCTC	198	NM_214201.1
GPX4	Glutathione peroxidase 4	GAGCTTTAGCCGCCTGTTC	GGTACTTGTCCAGGTTCACCA	176	NM_214407.1
BAX	BCL2 associated X	GCTGACGGCAACTTCAACTG	GCGTCCCAAAGTAGGAGAGG	202	NM_214285.1
CASP3	Caspase 3	TGGCATGTCGATCTGGTACA	CACGCAAATAAAACTGCTCCTT	170	NM_214131.1
CASP8	Caspase 8	GAGAATGTTGGAGGAAAGCAA	AAAAGCATGACCCTGTAGGC	165	NM_001031779.2
PPIA	Peptidylprolyl isomerase A	CTGAAGCATACGGGTCCTGG	CCAACCACTCAGTCTTGGCA	100	XM_021078519.1

qPCR was performed in a mixture containing 5 µL of iTaq Universal SYBR Green Supermix (Applied Biosystems, Foster City, California, USA), 1 µL of each primer pair (500 nM), 2 µL of cDNA and 1 µL of dH_2_O. All the reactions were carried out in duplicate in a QuantStudio 5 Real-Time PCR System (Applied Biosystems, Foster City, California, USA) with the following thermal cycling profile. Initial UDG activation at 50 °C for 2 min and an initial denaturation at 95 °C for 2 min. These steps were followed by 40 cycles of 5 s of denaturation at 95 °C and 30 s of annealing at 60 °C. A final melt curve analysis was performed to confirm the PCR specificity by the detection of a single distinct peak. The expression of PPIA was used to normalize the data. With the results obtained, the relative expression of each gene was calculated using the ΔΔCt method with efficiency correction^59^.

### Statistical analysis

The continuous variables are expressed as the mean ± standard deviation (SD). The mean percentage ± SD of the binary variables (cleavage, blastocyst rates, total efficiency and hatching rate) was obtained by calculating the percentage in every well of each group and in each replicate. The variables were analyzed to evaluate normality by the Kolmogorov–Smirnov or Shapiro–Wilk tests as appropriate. Groups with a success rate of 0% were excluded from the normality tests. The parametric variables were compared by mixed-model ANOVA followed by the Bonferroni post hoc test (Experiment 1) or by unpaired Student’s t test corrected for inequality of variances (Experiment 2). The nonparametric variables (hatching rates and TCN, Experiment 2) were analyzed by the Mann–Whitney U test. The statistical analysis was performed using the IBM SPSS 24.0 Statistics package (SPSS, Chicago, IL, USA). Differences were considered significant at *P* < 0.05.
